# Oil Spill Detection by SAR Images: Dark Formation Detection, Feature Extraction and Classification Algorithms

**DOI:** 10.3390/s8106642

**Published:** 2008-10-23

**Authors:** Konstantinos N. Topouzelis

**Affiliations:** Joint Research Centre (JRC), European Commission, Via Fermi 2749, 21027, Ispra (VA), Italy; E-mail: Kostas.Topouzelis@jrc.it

**Keywords:** Oil spill, sea pollution, SAR

## Abstract

This paper provides a comprehensive review of the use of Synthetic Aperture Radar images (SAR) for detection of illegal discharges from ships. It summarizes the current state of the art, covering operational and research aspects of the application. Oil spills are seriously affecting the marine ecosystem and cause political and scientific concern since they seriously effect fragile marine and coastal ecosystem. The amount of pollutant discharges and associated effects on the marine environment are important parameters in evaluating sea water quality. Satellite images can improve the possibilities for the detection of oil spills as they cover large areas and offer an economical and easier way of continuous coast areas patrolling. SAR images have been widely used for oil spill detection. The present paper gives an overview of the methodologies used to detect oil spills on the radar images. In particular we concentrate on the use of the manual and automatic approaches to distinguish oil spills from other natural phenomena. We discuss the most common techniques to detect dark formations on the SAR images, the features which are extracted from the detected dark formations and the most used classifiers. Finally we conclude with discussion of suggestions for further research. The references throughout the review can serve as starting point for more intensive studies on the subject.

## 1. Introduction

This review is primarily targeted to readers new to the use of SAR imagery for oil spill detection and who want to gain an overview of the problem without getting into complex details. It assumes a basic understanding of the principles of SAR and it benefits readers who wish to extend their general knowledge of SAR to the specific application.

Among the different types of marine pollution, oil is a major threat to the sea ecosystems. The source of the oil pollution can be located on the mainland or directly at sea. Sea-based sources are discharges coming from ships or offshore platforms. Oil pollution from sea-based sources can be accidental or deliberate. Fortunately, the number of marine accidents and the volume of oil released accidentally are on the decline. On the other side, routine tanker operations can lead still to the release of oily ballast water and tank washing residues. Furthermore, fuel oil sludge, engine room wastes and foul bilge water produced by all type of ships, also end up in the sea. In the last decade maritime transportation has been growing steadily. More ships also increase the potential number of illegal oil discharges. Both oil tankers and other kinds of ships are among the suspected offenders of illegal discharges.

The different tools to detect and monitor oil spills are vessels, airplanes, and satellites. Vessels, especially if equipped with specialised radars, can detect oil at sea but they can cover a very limited area. The vessel, however, remains necessary in case oil sampling is required. The main systems to monitor sea-based oil pollution are the use of airplanes and satellites equipped with Synthetic Aperture Radar (SAR). SAR is an active microwave sensor, which captures two dimensional images. The brightness of the captured image is a reflection of the properties of the target-surface. The possibility of detecting an oil spill in a SAR image relies on the fact that the oil film decreases the backscattering of the sea surface resulting in a dark formation that contrasts with the brightness of the surrounding spill-free sea. Spaceborne SAR sensors are extensively used for the detection of oil spills in the marine environment, as they are independent from sun light, they are not affected by cloudiness, they cover large areas and are more cost-effective than air patrolling.

**Figure 1.**
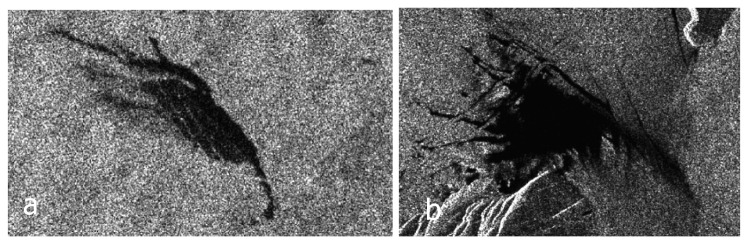
Two examples of dark formations: (a) Verified oil spill on a SAR image taken on 6 September 2005 close to Ancona, Italy. (b) Verified look-alike on a SAR image taken on 25 August 2005 close to Otranto, Italy (Adapted from Stathakis *et al.* [16]).

Radar backscatter values from oil spills are very similar to backscatter values from very calm sea areas and other ocean phenomena named “look-alikes” (e.g. currents, eddies). Figure 1 presents an example of a verified oil spill and a verified look-alike on a SAR image. Several studies aiming at oil spill detection have been conducted. The first comprehensive publications on the subject [1-5] concerned methodologies to distinguish oil spills from look-alikes. Solberg *et al.* [1] presented an automatic statistical approach while Del Frate *et al.* [2] used a neural network classifier. Espedal and Wahl [3] used wind history information to detect oil spills, Espedal *et al.* [4] focused on detection near offshore platforms and Fiscella *et al.* [5] used a probabilistic approach for detection and discrimination. The first reconnaissance study for the Mediterranean Sea using more that 1600 SAR images was given by Pavlakis *et al.* [6]. The second wave of interest on the subject came some years later, with new techniques and methodologies [7-14]. De Souza *et al.* [7] presented and intelligent system to extract features from oil slicks, Keramitsoglou *et al.* [8] an automatic system based on fuzzy logic, Karathanassi *et al.* [9] an object-oriented methodology, Mercier and Girard-Ardhuin [10] a classification method using kernel expansion and Ramalho - Medeiros [11] used boosting techniques. Topouzelis *et al.* [12] presented an updated study on discrimination using neural networks, Solberg and Brekke [13] summarized the detection techniques in Northern European waters while Serra-Sogas *et al.* [14] were focused in western Canadian waters. Finally, a detailed introduction to oil spill detection by satellite remote sensing is given by Brekke and Solberg [15].

Long term monitoring using SAR data was firstly performed by Pavlakis *et al.* [6, 17] for the Mediterranean basin and Gade and Alpers [18] for specific areas in Mediterranean Sea. The Joint Research Centre (JRC) continued these initiatives and monitored the European seas for the following years [19-23]. Bernardini *et al.* [19] focused on the Adriatic Sea, Ferraro *et al.* [20] on the French Environmental Zone, Ferraro *et al.* [21] presented an overview of the existing operational techniques in the Mediterranean Sea, Tarchi *et al.* [22] focused on the seas around Italy for the years 1999-2004 and Topouzelis *et al.* [23] on the Mediterranean basin for the years 1999-2002.

The Norwegian company Kongsberg Satellite Services (KSAT) in Tromsø, Norway, was one of the primary European providers of a satellite based oil spill detection service. Nowadays, the European Space Agency (ESA) through the Global Monitoring for Environment and Security (GMES) program has funded the MARCOAST project on Marine and Coastal surveillance to develop a harmonised service chain in Europe. Oil spill detection using spaceborne SAR data became operational in European waters form the middle of 2007 with the CLEANSEANET service of the European Maritime Safety Agency (EMSA). The service provides a range of detailed information including oil spill alerts to European Member States, rapid delivery of available satellite images and oil slick position.

## 2. SAR remote sensing sensors for oil spill detection

Several spaceborne SAR systems have been used for oil spill monitoring. They usually are characterized by their frequency (or band). The NASA's SEASAT satellite, which was launched in 1978, was the fist satellite designed to observe the sea surface with an L-band SAR system. Later, SAR systems were launched by the Russian Space Agency (RSA), the European Space Agency (ESA) and the Canadian Space Agency (CSA). The main satellites which were used or are in operational status for monitoring oil spills are presented in Table 1.

A SAR sensor can be described by the frequency band, the polarisation which refers to the geometry of the tip of the electric vector, the incidence angle i.e. the angular relationship between the radar beam and the ground target, the swath width i.e. width of the imaged scene and the image resolution i.e. size of the smallest detail identifiable on an image. There is a trade-off between the image resolution and the swath coverage. Usually, for oil spill detection, large swath widths are chosen at the expense of lower resolution. This approach is adopted because it is in our interest to cover as much area as possible even if very small oil spills can not be detected. Table 2 presents the satellite modes that are commonly used for sea monitoring above European waters.

## 3. SAR imaging of oil spills

Oil films decrease the backscattering of the sea surface resulting in a dark formation on SAR images. There are different mechanisms responsible for the sea surface radar backscattering, which strongly depend on the incidence angle of the radar sensor. In a quite large range of angles, approximately from 20° to 50°, the main agent of radar backscattering are the wind-generated short gravity-capillary waves. The oil film has a dampening effect on these waves locally decreasing the backscattering. It is implicitly assumed that a light wind field exists in order to activate short gravity-capillary waves. The minimum wind speed is in fact depending on the frequency of observation and the incidence angle. The most common radar sensors on board of operational satellites are using the C band. In this frequency range, a minimum wind field of 2–3 m/s creates sufficient brightness in the image and makes the oil film visible. On the other hand, when the wind speed is too high, it causes the spill to disappear. First, because the short waves receive enough energy to counterbalance the dumping effect of the oil film. Then, when the sea-state is fully developed, the turbulence of the upper sea layer may break and/or sink the spill or a part of it.

Several man made and natural ocean phenomena damp the wind generated short gravity – capillary waves. For this reason, some areas appear dark on SAR imagery in contrast to the surrounding sea. Any area on an image which is sufficiently darker than the neighbouring area can be characterized as a dark formation. It is particularly hard to determine how much darker an area has to be and there is not a clear criterion in the literature. Even in a single image, the degree of darkness and contrast required for the dark areas characterisation is not constant. Dark formations can be [24-25]: oil spills, low wind areas, organic film, fronts, areas sheltered by land, rain cells, current shear zones, grease ice, internal waves, upwelling zones, downwelling zones and eddies.

## 4. Methodologies for oil spill detection on SAR images

Two main approaches exist for the oil spill detection on SAR images: the manual approach, where operators are trained to analyse images for detecting oil spills and the semi-automatic or fully automatic approaches, where automatizations are inserted. Any formation on the image which is darker than the surrounding area has a high probability of being an oil spill and needs further examination. Although this process seems to be simple for a human operator, it contains three main difficulties for semi-automated or automated methods. First, fresh oil spills are brighter than older spills. They have a weak backscattering contrast relative to their surroundings and thus cannot be easily discriminated. Second, dark areas can have various contrast values, depending on local sea state, oil spill type, image resolution and incidence angle. Third, look-alike phenomena are presented as dark areas too.

### 4.1. Manual inspection

Manual inspection is the most popular technique for oil spill detection as is it not very complex and under certain circumstances can be easily reproduced. Nevertheless, it is not very reliable as it depends on the experience of the interpreter. In this approach operators are trained to detect oil spills through photo-interpretation. At fist stage all the possible candidates being oil spills are detected on a SAR image. Then a discrimination process is performed to distinguish oil spills from look-alikes. Some look-alikes are quite easy to classify as they have characteristic shapes and configurations completely different from those of the oil spills, such as dark patches caused by internal wave areas, eddies, or rain cells. However, a first sight analysis is not sufficient in complicated cases. The discrimination is particularly difficult in presence of natural oil slicks or areas with low wind speeds. In these situations a more detailed analysis is necessary, where several factors have to be taken into account. The most important are: the wind conditions, the period of the year, the shape analysis, the slick size and the general morphology of the observed area.

The knowledge of wind conditions is crucial, as low wind speeds of 2-3 m/sec result in many dark formations while with wind speeds above 8-10 m/sec the oil can not be detected. The period of the year is useful to discriminate natural slicks (i.e. algae bloom) and grease ice on summer, while the slick size is consider to exclude low wind areas or even large natural slicks. The general morphology of the observed area is crucial to distinguish dark formations caused by suddenly changes of the wind conditions i.e. the passage form a region in which the wind is not present to another in which wind is blowing. In many cases the dark formations are the result of the sheltering action due to area topography (e.g. areas close to the land, high submarine mountains, oil platforms). More complicated are the areas containing fronts, which are boundaries between water masses with dissimilar properties, like two water masses of different densities (due to different temperatures or/and salinities). In these cases dark formations can have extremely high combinations of shapes and sizes.

**Figure 2.**
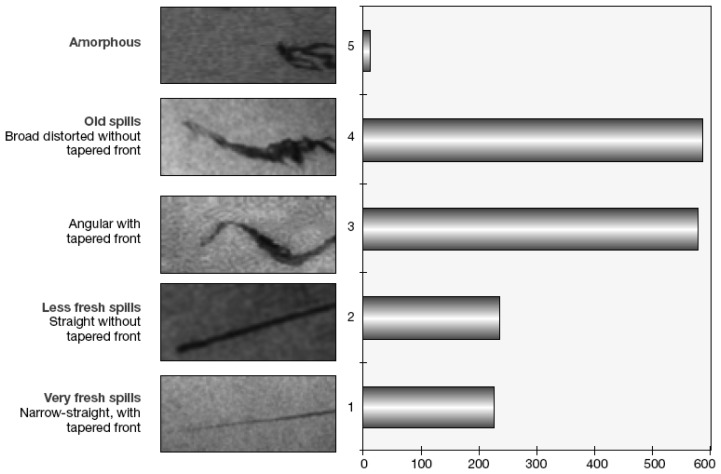
Classification of 1638 detected oil spills in terms of their shapes (adapted from Pavlakis *et al.* [6]).

Shape analysis is very useful for discriminating oil spills from look-alikes, mainly natural phenomena, since the two categories have specific characteristics. Shape analysis takes into consideration the characteristics of the border, the tails and the roundness of the dark formations. The borders of man-made spills are usually very well defined, with a sharp step in the backscattering values between the spilled region and the surrounding region. On the contrary, natural phenomena usually have more structured borders. However, old spills present much more complex border structure that a fresh one. The tails can be thin, straight or slightly curved for oil spills, while look- alikes present a “natural” behavior in the image with smoother turnings. Look-alikes can be some kilometers in length probably due to wind sheltering action and are usually connected with natural structures like eddies. Roundness of dark formations is essential for identifying fresh spills, elongated with or without curves. Usually man made spills are elongated, while many natural phenomena have round shape. Roundness can not really be measured, therefore is in generally based on the experience of the photo-interpreter. A very good categorization of the oil spills is given by Pavlakis *et al.* [6], where more than 1,600 oil spills are classified in five categories (Figure 2).

In general it can be assumed that dark formations are usually classified by photo-interpreters as potential oil spills according to the following criteria:

- Dark homogeneous spots in a uniform windy area;
- Linear dark areas, not extremely large, with abrupt turns i.e. most likely abrupt turns due to wind directions change or surface current. Natural slicks in these conditions tend to disappear. Man made slicks have higher viscosity and tend to change their shape.

Dark formations are usually classified by photo-interpreters as look-alikes according to the following criteria:

- Low wind areas;
- Coastal zones due to wind sheltering;
- Elongated dark areas with smooth turnings in spiral shape.

When oil spill detection service is provided with manual approach, usually an assignment of the possible oil spill is also provided. Mainly three categories are used representing slicks with high, medium or low probability of being oil. This assignment is mainly based on the experience of the photo-interpreters and is under discussion by the research community.

To this end the experience of the interpreter and especially its ability to apprehend the nature of the image manifestations, become a critical factor. As such experience is not widely available, efforts are made to develop systems, which will detect and identify dark formations as oil spills in an automatic or in a semi-automatic way.

### 4.2. Semiautomatic and fully automatic methodologies

**Figure 3.**
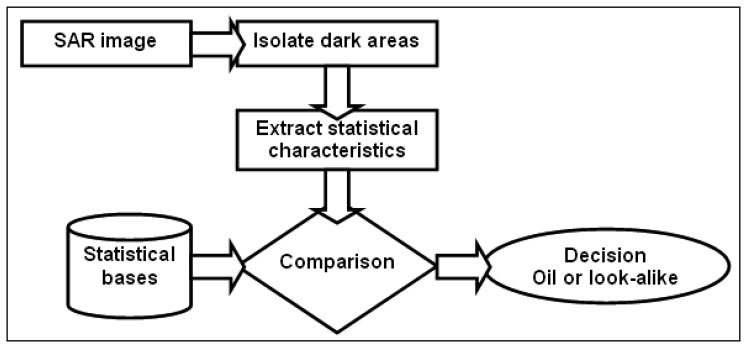
The basic functions of oil spill detection methodologies.

Semiautomatic and fully automatic methodologies are not very popular for oil spill detection as they are complex, they can not be easily reproduced and require specific knowledge on image understanding, pattern recognition and classifications theories. The basic idea for such approaches is presented in figure 3 and can be summarized in four steps [1, 6]:

1. Detection and isolation of all dark formations presented in the image. Mainly this step is a result of thresholding and segmentation processing.
2. Extraction of statistical parameters of the dark formations, so called “features” for each oil spill candidate. These features are related with the geometry of the formation (e.g. area, perimeter) their physical behavior (e.g. mean backscatter value) and their context in the image (e.g. distance to ships).
3. Test of the extracted values against predefined values, which characterize man-made oil spills and look-alike phenomena. These values are usually determined through phenomenological considerations and statistical assessments.
4. Classification of the dark formations to oil spills or look-alikes. Several classifiers have been used, i.e. statistical approach through computation of probabilities, neural networks, fuzzy logic, etc.

Semi-automatic or fully automatic algorithms should operate under all wind conditions, including low winds where high number of look-alikes is expected. In these conditions the false alarm ratio (i.e. look-alikes that categorized as oil spills) is usually extremely high for the automatic algorithms.

#### 4.2.1. Dark formation detection

Dark formation detection is considered the fundamental step in oil spill detection systems and constitutes the first step in oil spill detection approaches. Several techniques have been presented in bibliography for detecting dark formations in SAR images. An overview of them is given in the following paragraphs. Once dark formations are detected, classification methods are applied to characterise them as oil spills or look-alike objects. If dark formations are not detected in this step they will never be classified.

Dark formations can be located manually by cropping a broader area containing the dark formation, or an image window with fixed size can be used, in which threshold algorithms -adapted or not- can be applied. Simple thresholds have one value for the whole image e.g. the half of the average Normalized Radar Cross Section (NRCS) of the image [5], or NRSC minus the standard deviation [28]. In adaptive algorithms threshold is calculated locally, mainly on areas covered by a moving window. In Solberg *et al.* [1, 29] the threshold is set k dB below the mean value of the moving window and it is calculated using a multiscale pyramid approach and a clustering step. In Karathanassi *et al.* [9], the threshold is fully adaptive to local contrast and brightness of large image segments, therefore the image window does not have a fixed size but it varies according to brightness and contrast values of large areas in the image. Del Frate *et al.* [2], used an edge detection technique based on image histograms which were derived from areas with suspicious dark formations. Kannaa *et al.* [30] applied a hysteresis thresholding [31] where linear dark formations were successfully detected. Huang *et al.* [32] applied a partial differential equation (PDE) - based level set technique, which represents the slick surface as in implicit propagation interface.

All the above studies use statistical based techniques to locate dark formations. A different approach is given by Liu *et al.* [33], Wu and Liu [34] were the use of wavelets for oil spill detection was described. This study was performed for ocean feature detection on SAR data including oil spills. In later studies [35, 36] wavelets were used specific for the oil spill detection problem. Moreover, in Benelli and Garzelli [27], dark formations were detected using a fractal dimension estimation, where a multi resolution algorithm based on fractal geometry for texture analysis was applied. Later, Marghany *et al.* [37] presented a method for modification of the formula of the fractal box counting dimension. A different technique, based on texture analysis, was presented by Marghany [38, 39] where several textures (i.e. entropy, homogeneity, contrast, energy and correlation) were examined to detect dark formations. In Topouzelis *et al.* [40] an investigation of neural network capabilities and constraints to successfully detect dark formations using high resolution SAR images was performed.

All the above mentioned techniques have one common goal. To detect dark formations on SAR images by means of their position and shape. The next step is to extract several features, which describe the black formation in order to use them as input to the classifier.

#### 4.2.2. Feature extraction

Features are very important for the classification because they are used as inputs to the classifier. Therefore, the combination of features which discriminate better the oil spill from the look-alikes is of very high importance for the classifier and for the method's accuracy. In general oil spill detection methodologies traditionally use arbitrary selected quantitative and qualitative statistical features for classifying dark objects on SAR images into oil spills or look-alike phenomena.

The features which usually used for oil spill detection can be generally grouped in three major categories [9, 15, 16]. Features referring to the geometrical characteristics of oil spills (e.g. area, perimeter, complexity), features capturing the physical behavior of oil spills (e.g. mean or max backscatter value, standard deviation of the dark formation or a bigger surrounding area) and features referring to the oil spill context in the image (e.g. number of other dark formations in the image, presence of ships).

The absence of a systematic research on features extracted as well as their contribution to the classification results force researchers to arbitrary select features as input to their systems. Solberg *et al.* [1] used 11 features, Fiscella *et al.* [5] used 14, in general different from the 11 used by Del Frate *et al.* [2]. Keramitzoglou *et al.* [8] used 14 and Karathanassi *et al.* [9] used 13 features many of them different from the previous studies.

The lack of systematic research can be attributed to the fact that the existing methodologies for searching into a large number of different compilations have not been fully exploited. Stathakis *et al.* [16] and Topouzelis *et al.* [41] tried to bridge this chasm and to discover the most useful features of oil spill detection using a combination of genetic algorithms and neural networks.

Several studies try to unify all the features used having similar characteristics [15, 42, 43]. Table 3 presents a grouping of the 25 most commonly used features applied in the majority of research studies. The first six features (1-6) refer to the geometrical characteristics, the next sixteen features (7-22) refer to the physical characteristics and the last three (23-25) to the texture characteristics of the dark formations. Detailed description can be found at Stathakis *et al.* [16] and Topouzelis *et al.* [41].

#### 4.2.3. Classifiers

The purpose of the classifier is to distinguish oil spills from look-alikes. How difficulty is the classification task depends on the variability of the oil spill and look-alike examples. Classifiers learn the patterns from examples (training step) and in a later stage are called to take a decision (classification step). The most known are the statistical classifiers, in which the classification decision is based on probability. Statistical classifiers are quite popular as they are rather simple, reliable and can be easily reproduced. Their classification reliability can be measured *a priory*, according to the database samples of oil spills and look-alikes.

Solberg *et al.* [1] proposed a statistical modeling with a rule based approach. The probabilities assigned using Gaussian density function and derived from a signature database of 7,051 dark formations containing 71 oil spills and 6,980 look-alikes. These dark formations were extracted from 84 ERS SAR images, from which 36 did not contain any oil spills. The method correctly classified 94% (67 out of 71) of oil spills and 99% (6,905 out of 6,980) of look-alikes using a leave-one-out approach. In a recent study [29] an updated version of the method was presented for Radarsat and Envisat images. Their training dataset consisted from 56 Envisat WS ASAR images and 71 Radarsat SAR images while the reported test set consisted of 27 Envisat images containing 37 oil spills and 12110 look-alikes. The method had an accuracy of 78% in oil spill classification (29 out of 37) and of 99% in look-alike classification (12,033 out of 12,110).

A similar statistical classification methodology was presented by Fiscella *et al.* [5]. They applied a Mahalanobis and a compound probability classifier. Their training set was 80 oil spills and 43 look-alikes and they measured the probability *p* of dark formations to be oil spill (*a priory* classification probability). They used three classification categories: oil spills, uncertain and look-alikes. The percentage of the total data correctly classified for the Mahalanobis classifier with p>2/3 (i.e. three classification categories) was 78% and with p>1/2 (i.e. only oil spills and look-alikes) was 83%. For compound probability classifier with p>2/3 was 79% and with p>1/2 was 82%. The test set contained 21 dark formations of which 11 oil spills, 4 uncertain and 6 look-alikes. Mahalanobis classifier corresponded correctly at 71% of the cases and a compound probability classifier at 76% of the cases.

Nirchio *et al.* [28] presented another statistical approach based on multi regression analysis (or Fisher discrimination approach). They used 13 features as inputs and tried to set up a relation between the predictor variables and the dependent variable on a dataset contained 153 verified oil spills and 237 look-alikes. They reported an *a priory* percentage of correct classification higher than 90% on the training dataset. For the testing dataset they used 14 images, in which 31 oil spills were present. Their method detected correctly 23 of them i.e. 74% oil spill detection accuracy.

A different classification methodology was presented by Del Frate *et al.* [2]. They used neural networks to classify the dark formations. Neural network classifiers are not very popular as they are rather complex and they require specific knowledge on the theory of neural systems. Their complexity relates with decisions on network family, network architecture, on the way the data are introduced during the training and the point where the training should stop. They considered reliable classifiers since they have the ability to learn during training.

Del Frate *et al.* [2] applied the Multilayer Perceptron (MLP) network family with a topology of 11 inputs (i.e. the calculated features describing the dark formations), one output and two hidden layers with 8 and 4 neurons respectively. Their data set was 600 ERS images in low resolution from which they extracted 139 dark formations 71 oil spills and 68 look-alikes. Using the leave-one-out approach the method misclassified 18% of the oil spills and 10% of the look-alikes.

Neural networks were used also as classifiers for oil spill detection by Topouzelis *et al.* [12]. They used a MLP network with topology 10:51:2, i.e. 10 features as inputs, 51 neurons in the hidden layer and 2 output nodes. The topology and the input features were chosen using a genetic algorithm. The genetic algorithm had chosen the selected 10 from a base of 25 inputs and the proper topology after searching 100 generations using 7 bit chromosome. Detailed information regarding the selected topology and the feature selection is given by Stathakis *et al.* [16] and Topouzelis *et al.* [41]. Their dataset consisted of 24 high resolution SAR images containing 159 dark formations, 90 look-alikes and 69 oil spills. They randomly split the available data set into equally sized parts one for training and one for testing. The oil spills accuracy reported on the test data was 91% (detected 31 oil spills out of 34) and the look-alike 87% (detected 39 of out 45).

Another classification methodology, based on fuzzy logic, was implemented by Keramitsoglou *et al.* [8] and by Karathanassi *et al.* [9]. Fuzzy classifiers work with ranges of values, solving problems in a way that more resembles human logic. The input variables in a fuzzy based methodology are mapped into by sets of membership functions. They require specific knowledge on the theory of fuzzy systems but they considered reliable classifiers since they try to resemble human logic and they can be reproduced easily.

Keramitsoglou *et al.* [8] estimated the probability of a dark formation to be oil spill using an artificial intelligence fuzzy modeling system. Their method developed using 9 ERS-1/2 low resolution images and was tested on 26 images. Five features used as inputs, in general different from those used before. The method responded perfectly on 23 of 26 images, resulting in an overall performance of 88%.

Karathanassi *et al.* [9] also proposed a classification method based on fuzzy classification rules. Their methodology was based on an object oriented image classification technique, in which on the first step homogeneous image objects are extracted in any chosen resolution and in later stage are classified by means of fuzzy logic. They used 13 features as inputs on 12 ERS-1/2 high resolution images. They reported 99% overall performance.

Comparison between the different classifiers in terms of classification accuracies is very difficult. Mainly because oil spill detection approaches use different data sets, have different dark formation detection techniques, extract arbitrary number of features and in the end use different classifiers. Therefore, the reported classification accuracies can not be directly compared. Table 4 presents the most used oil spill detection approaches and where available the number of the satellites images used, their resolution, the dark formation method, the number of the calculated features and finally their accuracies.

Another issue with high importance for the detection methodologies is the computational time. Unfortunately, there are no sufficient data in the literature to compare the methodologies in terms of necessary time from image acquisition to final classification – report. This comparison should be made under the same data in order to check not only the time frame, but also the methods accuracy. Nevertheless, we can point out that as long as the methodologies have been developed, the most time consuming step for analyzing a new image is the dark formation detection step. Once the dark formations have been detected, the feature calculation step and the classification step need some seconds to complete their actions.

## 5. Discussion and conclusions

The capability of space-borne SAR sensors to detect oil spills over the sea surface is well known and proven. The possibility of using space radar imagery for long term monitoring of operational oil pollution at basin scale has also been demonstrated. Moreover, operational service on oil spill detection in European waters is provided by EMSA and new SAR sensors are scheduled to be launched in the following years. SAR is the most applicable sensor for operational oil spill detection as it covers wide areas and operates at all-weather, day and night.

As the quantity of the SAR data increases rapidly there is a big need for semi or fully automatic methodologies to detect and identify dark formations as oil spills, fast and accurate. There are several methodologies proposed in the literature but their results are under discussion and they cannot really be compared. The main reason is that each study is using its available data set, which in most of the cases does not contain verified examples. It is worth mentioning that only one of the methodologies presented in Table 4 used verified data. Therefore, there is a need for a common database with verified oil spills and look-alikes which will be widely available in the scientific community. This database should contain a wide range of verified oil spills (in age, shape and brightness) and verified look-alikes, in different wind conditions, by several SAR sensors, polarizations and modes. Also, a categorization is needed for the verification means (e.g. airplane, vessel) and the time gap between the image acquisition and the verification. Only then the different methodologies will be comparable and their improvements measurable. Moreover, under this perspective the three main parts of the methodologies (i.e dark formation detection, feature extraction and classification) can also be examined and compared in terms of accuracy and processing time.

The key issues for an effective oil spill methodology are given by Kubat *et al.* [26]. They refer to five critical factors which should be taken into account; the scarcity of the data in terms presence of oil spills, the imbalanced training set (there are more negative examples i.e. look-alikes that positive examples i.e. oil spills), the validity of the data selection (there is no guaranty that the examples used at the development phase are representatives of the examples that will arise after development), the feature selection procedure and the highly dynamic environment in terms of dataset, feature selection and classification algorithm. Another issue is the accuracy estimator, i.e. the performance of a method can be also added as a key factor. These issues have to be very carefully studied before designing a new methodology or applying a new dataset to existing methods.

Semi automatic methods can be very helpful under certain circumstances but the manual approach will never be eliminated and more work is needed on the comparison versus semi and full automatic methods. In particular predefined automatic methods should be compared against photo-interpreters with different level of experience. This test will help to better understand the advantages and the limitation of the automatic methods. Last but not least, current automatic methodologies should put more effort to their weak points especially on detection at low wind situations and where natural films are present.

Nowadays, Europe is using SAR data as a powerful tool to verify at different time and space scales the variations of the sea based pollution. These variations should result in specific actions and new measures to further extend and reinforce the level of protection of the marine environment against oil pollution.

## Figures and Tables

**Table 1.** Satellites carrying SAR instruments focusing in ocean observation.

**Satellite (sensor)**	**Operative**	**Owner**	**Band**
SEASAT	1978 – 1978	NASA	L
ALMAZ	1991 – 1992	RSA	S
ERS-1	1991 – 1996	ESA	C
ERS-2	1995 – operating	ESA	C
RADARSAT-1	1995 – operating	CSA	C
RADARSAR-2	2007– operating	CSA	C
ENVISAT (ASAR)	2002 – operating	ESA	C
ALOS (PALSAR)	2006 – operating	JAXA	L
TerraSAR-X	2007 – operating	DLR	X
Cosmos Skymed-1/2	2007 – operating	ASI	X

ASI – Italian Space Agency, DLR - German Aerospace Centre, ESA – European Space agency, JAXA - Japan Aerospace Exploration Agency, NASA - National Aeronautics and Space Administration (USA).

**Table 2.** Examples of satellite modes (adapted from Brekke and Solberg [15]).

**SAR sensor**	**Mode**	**Resolution (m)**	**Pixel Spacing (m)**	**Swath width (Km)**	**Incidence angle (**°**)**
ERS-2	PRI	30 × 26.3	12.5 × 12.5	100	20 -26
ENVISAT	IM	30 × 30	12.5 × 12.5	100	15 - 45
RADARSAT-1	SCN	50 × 50	25 × 25	300	20 - 46
RADARSAT-1	SCW	100 × 100	50 × 50	450 – 500	20 - 49
ENVISAT	WSM	150 × 150	75 × 75	400	16 - 44

PRI – Presision Image Mode, IM – Image Mode, SCN – ScanSar Narrow, SCW -ScanSar Wide, WSM - Wide Swath Mode

**Table 3.** Commonly features used (adapted from Stathakis *et al.* [16]).

**No**	**Features**	**Code**
1	Area	A
2	Perimeter	P
3	Perimeter to area ratio	P/A
4	Complexity	C
5	Shape factor I	SP1

6	Shape factor II	SP2
7	Object mean value	OMe
8	Object standard deviation	OSd
9	Object power to mean ratio	Opm
10	Background mean value	BMe

11	Background standard deviation	BSd
12	Background power to mean ratio	Bpm
13	Ratio of the power to mean ratios	Opm/Bpm
14	Mean contrast	ConMe
15	Max contrast	ConMax

16	Mean contrast ratio	ConRaMe
17	Standard deviation contrast ratio	ConRaSd
18	Local area contrast ratio	ConLa
19	Mean border gradient	GMe
20	Standard deviation border gradient	GSd

21	Max border gradient	GMax
22	Mean Difference to Neighbors	NDm
23	Spectral texture	TSp
24	Shape texture	TSh
25	Mean Haralick texture	THm

**Table 4.** Several oil spill detection approaches and their characteristics.

**#**	**Method**	**Images and/or resolution**	**Preprocessing**	**Dark Formation detection method**	**Number of features**	**Dark formations**	**Results [method of evaluation]**
1	Probabilistic approach (statistical modeling with a rule based approach)	ERS-1, 84 images	a) Calibration	Adaptive threshold (multiscale pyramid approach and a clustering step)	11	7051 dark, formations, 71 oil spills, 6980 lookalikes	94% oil spills class. acc. 99% look-alikes class. acc. [leave-one-out approach]
2	Neural Network (MLP 11:8:4:1)	ERS, 600 low resolution images	a) Resampling, b) Radiometric range correction c) Georeference	Adaptive threshold (Edge detection based on histogram of areas with dark formations)	11	139 dark formations, 71 oil spills, 68 lookalikes	82% oil spills class. acc. 90% look-alikes class. acc. [leave-one-out approach]
3	Probabilistic approach (mahalanobis classifier, compound probability classifier)	ERS, Low resolution for inspection and high in case of processing		Simple threshold (image statistical value i.e. average intensity value)	14	Training set: 123 dark formations, 80 oil spills, 43 look-alikes Testing set: 21 dark formations, 11 oil spills, 4 uncertain, 6 look-alikes	Mahalanobis: 82% oil spills class. acc. 0% uncertain class. acc. 100% look- alikes class. acc [test set] compound probability: 91% oil spills class. acc. 50% uncertain class. acc. 67% look- alikes class. acc. [test set]
4	Probabilistic approach. (multi regression analysis)	ERS-1/2, high resolution. 14 for testing	a) Calibration b) Incidence angle correction c) Land masking	Simple threshold (image statistical values i.e. average intensity value and standard deviation)	13	Training set: 390 dark formations, 153 oil spills 237 look-Alikes Testing set: 31 oil spills	A *priori* percentage of correct classification 90% on training set. 74% oil spill class. acc. [test set]
5	Fuzzy classification	ERS-1/2, 12 high resolution	a) 8-bit transformation b) Filtering	Adaptive threshold (local contrast and brightness of large image segments)	13		Overall performance 99%
6	Fuzzy classification	ERS-1/2, low resolution.9 for training, 26 for testing	a) Georeference b) Land masking c) Filtering	Adaptive threshold (local average intensity value and sTable factor)	5		Overall performance 88% [test set]
7	Neural Network (MLP 10:51:1)	ERS-2, 24 high resolution	a) 8-bit transformation b) Filtering c) Normalization	Neural network (MLP 1:3:1)	10	Training set: 35 oil spills, 45 look- alikes Testing set: 34 oil spills, 45 look- alikes	91% oil spills class. acc. 87% look-alikes class. acc. [test set]
8	Probabilistic approach (statistical. Modeling with a rule based approach)	Training 71 Radarsat 56 Envisat Testing: 27 Envisat	a) Land masking b) Calibration	Adaptive threshold (multiscale pyramid approach and a clustering step)	13	Testing set: 37 oil spills 12110 lookalikes	78% oil spill class. acc. 99% lookalike class. acc. [test set]

1: Solberg *et al.* [1], 2: Del Frate *et al.* [2], 3: Fiscella *et al.* [5], 4. Nirchio *et al.* [28], 5: Karathanassi *et al.* [9], 6: Keramitsoglou *et al.* [8], 7. Topouzelis *et al.* [12], 8: Solberg *et al.* [29].
